# GSK-3β/NFAT Signaling Is Involved in Testosterone-Induced Cardiac Myocyte Hypertrophy

**DOI:** 10.1371/journal.pone.0168255

**Published:** 2016-12-15

**Authors:** Javier Duran, Cesar Oyarce, Mario Pavez, Denisse Valladares, Carla Basualto-Alarcon, Daniel Lagos, Genaro Barrientos, Mayarling Francisca Troncoso, Cristian Ibarra, Manuel Estrada

**Affiliations:** 1 Laboratorio de Endocrinología Celular, Programa de Fisiología y Biofísica, Instituto de Ciencias Biomédicas, Facultad de Medicina, Universidad de Chile, Santiago, Chile; 2 Programa de Anatomía y Biología del Desarrollo, Instituto de Ciencias Biomédicas, Facultad de Medicina, Universidad de Chile, Santiago, Chile; 3 Heart Failure Bioscience Department, Cardiovascular and Metabolic Diseases (CVMD), Innovative Medicines & Early Development iMED Biotech unit, AstraZeneca R&D, Mölndal, Sweden; Maastricht University, NETHERLANDS

## Abstract

Testosterone induces cardiac hypertrophy through a mechanism that involves a concerted crosstalk between cytosolic and nuclear signaling pathways. Nuclear factor of activated T-cells (NFAT) is associated with the promotion of cardiac hypertrophy, glycogen synthase kinase-3β (GSK-3β) is considered to function as a negative regulator, mainly by modulating NFAT activity. However, the role played by calcineurin-NFAT and GSK-3β signaling in testosterone-induced cardiac hypertrophy has remained unknown. Here, we determined that testosterone stimulates cardiac myocyte hypertrophy through NFAT activation and GSK-3β inhibition. Testosterone increased the activity of NFAT-luciferase (NFAT-Luc) in a time- and dose-dependent manner, with the activity peaking after 24 h of stimulation with 100 nM testosterone. NFAT-Luc activity induced by testosterone was blocked by the calcineurin inhibitors FK506 and cyclosporine A and by 11R-VIVIT, a specific peptide inhibitor of NFAT. Conversely, testosterone inhibited GSK-3β activity as determined by increased GSK-3β phosphorylation at Ser9 and β-catenin protein accumulation, and also by reduction in β-catenin phosphorylation at residues Ser33, Ser37, and Thr41. GSK-3β inhibition with 1-azakenpaullone or a GSK-3β-targeting siRNA increased NFAT-Luc activity, whereas overexpression of a constitutively active GSK-3β mutant (GSK-3βS9A) inhibited NFAT-Luc activation mediated by testosterone. Testosterone-induced cardiac myocyte hypertrophy was established by increased cardiac myocyte size and [^3^H]-leucine incorporation (as a measurement of cellular protein synthesis). Calcineurin-NFAT inhibition abolished and GSK-3β inhibition promoted the hypertrophy stimulated by testosterone. GSK-3β activation by GSK-3βS9A blocked the increase of hypertrophic markers induced by testosterone. Moreover, inhibition of intracellular androgen receptor prevented testosterone-induced NFAT-Luc activation. Collectively, these results suggest that cardiac myocyte hypertrophy induced by testosterone involves a cooperative mechanism that links androgen signaling with the recruitment of NFAT through calcineurin activation and GSK-3β inhibition.

## Introduction

Cardiac hypertrophy is an adaptive mechanism of the heart that enhances cardiac output in response to several physiological and pathological conditions [1]. In cardiac myocytes, this phenomenon is characterized by increases in cell size and protein synthesis and the re-expression of various fetal genes [2]. The development of hypertrophy in cardiac myocytes depends on the interaction between several intracellular signaling pathways related to cell growth [1, 3]. Testosterone, the main physiological anabolic/androgenic steroid hormone, induces cardiac hypertrophy *in vivo* and *in vitro* [4–7]. Testosterone exerts most of its biological effects by directly binding to the intracellular androgen receptor (AR), which acts as transcriptional activator [8]. Furthermore, testosterone also activates intracellular signaling pathways and thus triggers their multiple cellular effects [9, 10].

Determining the signaling pathways modulated by testosterone is critical because normal testosterone concentrations are necessary for multiple biological and physiological actions, including the maintenance of cardiac myocyte health, and changes (increases and decreases) in plasma testosterone concentrations are associated with elevated cardiovascular risk [11, 12]. However, androgens are also currently recognized to potentially produce additional beneficial cardiovascular effects by relaxing the vascular bed, reducing after-load, and rapidly increasing cardiac contractility, and this improves cardiac output and heart function [13]. Testosterone induces tissue-specific actions by modulating signaling pathways and gene expression. In addition, increased androgen receptor expression has been reported in skeletal muscle hypertrophy induced by androgens. However, the signaling pathways that control the hypertrophic actions of testosterone in cardiac cells remain to be elucidated.

Multiple factors regulate cardiac myocyte hypertrophy, and a substantial amount of evidence indicates that both nuclear factor of activated T-cells (NFAT) and glycogen synthase kinase-3β (GSK-3β) play predominant roles in the control of cardiac myocyte growth in response to pro-hypertrophic stimulation [14, 15]. Transcription factors of the NFAT family are composed of three functional domains: 1) the REL-homology domain that allows the association of NFAT-family proteins with other transcription factors, such as AP-1 [16]; 2) the NFAT-homology region (NHR), which contains nuclear localization (NLS) and nuclear export sequences [17]; and 3) a transcriptional activation domain, which recruits other transcriptional coactivators [18]. Under basal conditions, NFAT remains phosphorylated at serine residues in the conserved N-terminal region, and these residues mask the NLS and thus retain NFAT in the cytoplasm and prevent its nuclear migration and subsequent effect on gene expression. However, upon activation, NFAT is dephosphorylated, allowing it translocation to the nucleus. Dephosphorylation of the NFAT isoforms c1–c4 has been reported to depend on the phosphatase activity of calcineurin [19]. A recent interesting report determined that treatment of skeletal muscle cells with nandrolone, a 19-nor-testosterone-derivate, increases calcineurin-NFAT signaling to induce cell growth during atrophy caused by denervation [20]. Moreover, in the human prostate cancer cell line LNCaP, NFAT inhibition prevents the expression of AR-responsive genes involved in growth and metabolism [21]. Therefore, increasing evidence supports a role for NFAT signaling in the androgens actions on cellular growth.

Conversely, GSK-3β is a key anti-hypertrophic factor in cardiac cells [15, 22, 23] that regulates both the nuclear residence and the activity of NFAT [24]. In the nucleus, GSK-3β can phosphorylate the conserved serine residues located in the N-terminal domain of NFAT and thereby promote NFAT nuclear export and consequently control the transcriptional actions of NFAT [25]. Under basal conditions, GSK-3β is constitutively active, but under hypertrophic stimulation, GSK-3β is phosphorylated at Ser9, which inhibits its activity [26]. Different upstream pathways regulate the phosphorylation of GSK-3β at Ser9, including the phosphatidylinositol-3 kinase/Akt (PI3K/Akt) and extracellular signal-regulated kinases 1/2 (MEK/ERK1/2) [26–29]. GSK-3β has been described as a negative regulator of cardiac hypertrophy induced by endothelin-1 [30], insulin-like growth factor 1 [31], β-adrenergic agonists, and pressure overload [15, 32]. Furthermore, in mice, overexpression of a mutant GSK-3β, GSK3βS9A, which is unresponsive to Akt-dependent phosphorylation, was found to improve cardiac function [33] and prevent cardiac hypertrophy induced by insulin-like growth factor 1 [31, 34]. Moreover, GSK-3β induces β-catenin ubiquitination by phosphorylating it at Ser33, Ser37, and Thr41, which results in β-catenin protein destabilization and intracellular degradation through the proteasome pathway [35].

Here, we investigated whether testosterone-induced cardiac hypertrophy involves NFAT activation. Our results show that testosterone increases NFAT activity through calcineurin activation and GSK-3β inhibition. In addition, AR signaling modulates the transcriptional activity of NFAT, which contributes to the development of cardiac myocyte hypertrophy.

## Materials and Methods

### Reagents

The following reagents were purchased: Testosterone enanthate, FK506, cyclosporine A (CsA), 5-bromo-2-deoxyuridine (BrdU), bicalutamide, and cyproterone from Sigma-Aldrich (St. Louis, MO, USA); 1-azakenpaullone (1-Azk), 11R-VIVIT, PD98059, LY-294002, and Akt-inhibitor VIII from Calbiochem (La Jolla, CA, USA); CellTracker Green (5-chloromethylfluorescein diacetate) from Molecular Probes/Thermo Fisher Scientific (Eugene, OR, USA); and [^3^H]-leucine from NEN Radiochemicals Perkin Elmer (Waltham, MA, USA). All other reagents were of analytical grade and commercially available. Testosterone was diluted in absolute ethanol, the final concentration of which was 0.01% (v/v) in the stimulation medium; at this concentration, ethanol exerts no effect on biochemical determinations [10].

### Primary culture of neonatal rat cardiac myocytes

All animal-use procedures and protocols were approved by Institutional Animal Care and Use Committee (Faculty of Medicine, University of Chile, CBA # 0334 and 0768 FMUCH), in accordance with the National Institutes of Health Guide for the Care and Use of Laboratory Animals. Neonatal rat ventricular cardiac myocytes were isolated from the hearts of 1–3-day-old Sprague-Dawley rats as described previously [36]. The protocol used yields primary cultures of cardiac myocytes that are at least 90% pure [6]. To prevent fibroblast proliferation, the growth medium was supplemented with 25 μM BrdU. Cardiac myocytes were cultured in growth medium containing DMEM:M-199 (4:1) supplemented with 10% fetal bovine serum and 1% penicillin-streptomycin.

### Plasmids and transfection

A reporter plasmid was used to evaluate the transcriptional activity of NFAT; this plasmid contains NFAT-binding boxes cloned upstream of the firefly luciferase reporter gene (NFAT-Luc, from Dr. T. Finkel, Laboratory of Molecular Biology, NIH, Bethesda, MD, USA; Addgene plasmid #10959). To examine NFAT/GSK-3β signaling, cardiac myocytes were additionally co-transfected with a plasmid expressing a constitutively active form of GSK-3β (GSK-3βS9A) or a wild-type isoform of GSK-3β (GSK-3βWT) (from Dr. J. Woodgett, Mount Sinai Hospital, Toronto, Canada; Addgene plasmids #14754 and #13753, respectively). A plasmid constitutively expressing *Renilla* luciferase was used as the control. Transfections were performed using Lipofectamine 2000 (Invitrogen, Carlsbad, CA, USA), according to manufacturer specifications. In each experimental condition, plasmid DNA was used at a final concentration of 1 μg/10^6^ cells. The cells were stimulated with testosterone in the presence or absence of inhibitors. Next, the activities of both NFAT-Luc and *Renilla* luciferase were determined using the dual-luciferase kit Assay Reporter System (Promega, Madison, WI, USA) in a luminometer (Berthold Luminometer F12, Germany). The obtained data are expressed as the ratio of the NFAT-Luc luminescence to *Renilla* luminescence. Moreover, cells were transfected with siRNA targeting GSK-3β (siRNA-GSK-3β) (OriGene Technologies Inc., Atlanta, GA, USA) and siRNA targeting AR (siRNA-iAR) Santa Cruz Biotechnology, Santa Cruz, CA, USA) to reduce the protein expression levels. Non-targeting siRNA and non-transfected cells were used as controls in each condition. The siRNAs (20 nM) were transfected using Lipofectamine, and protein downregulation was confirmed through Western blotting (S1 and S2 Figs)

### Western blotting

Cells were cultured in 60-mm culture plates and serum starved for 12 h before testosterone stimulation. The experimental procedures for the preparation of total protein cell lysates and Western blotting have been described previously [10]. Briefly, 10–20 μg of proteins were separated using SDS-PAGE and transferred to nitrocellulose membranes, which were blocked in 5% milk-Tris-buffered saline (TBS)-0.05% Tween 20 (TBST) and then probed with the following primary antibodies: anti-phospho-GSK-3β (1:1,000), anti-GSK-3β (1:2,000), anti-phospho-β-catenin (1:1,000), and anti-β-catenin (1:1,000), Cell Signaling Technology (Boston, MA, USA); anti-AR (1:1,000), Santa Cruz Biotechnology; and anti-β-actin (1:5,000), Sigma-Aldrich (used as a control for protein loading in gels). Next, the membranes were washed three times in TBST, incubated with peroxidase-conjugated anti-rabbit or anti-mouse secondary antibodies (1:2,000; Pierce Thermo Scientific) for 1 h at room temperature, and washed thrice more in TBST. The protein bands in the membranes were visualized using the ECL Westar Supernova detection kit (Cyanagen, Italy) and band intensities were determined through densitometry performed using Image J software (NIH, Bethesda, USA).

### Cell size determination

Cardiac myocytes were cultured for 24 h in 12-well culture plates and transfected with plasmids or siRNAs. Next, culture medium was replaced with medium supplemented with testosterone (100 nM) alone or in the presence of inhibitors of either NFAT or GSK-3β and then cultured for an additional 48 h. To quantify changes in cell size induced by the hormone, the cardiac myocytes were incubated with the vital fluorescent probe CellTracker Green for 30 min. Images were acquired by confocal microscopy (40X, 1.4 NA objective; LSM Carl Zeiss Pascal 5, Oberkochen, Germany) and then analyzed using Image J software. At least 8 different fields from 4 independent cultures were examined for each condition (>80 cells).

### Amino acid incorporation

After 24 h of culture, control cardiac myocytes and myocytes transfected with siRNA-GSK-3β or GSK-3βS9A or pretreated with inhibitors were incubated with [^3^H]-leucine (2.5 μCi/mL) for additional 48 h in the presence or absence of testosterone. Next, the cells were washed 4 times with ice-cold PBS and treated with 10% trichloroacetic acid at 4°C for 1 h. The samples were centrifuged for 20 min at 15,000 RPM, and the pellets were washed once with ice-cold absolute acetone and dissolved in 0.2 M NaOH [37]. Aliquots from triplicate samples were counted in a liquid scintillation counter (Beckman Instruments, Fullerton, CA, USA). The data presented herein correspond to the ratio (basal counts/min)·μg^-1^ protein with respect to each experimental condition.

### Statistical analysis

The results are expressed as the mean ± SEM. To compare the difference between baseline and post-stimulation values, we performed *t* tests and analysis of variance (ANOVA) with Tukey’s post-test; p < 0.05 was considered statistically significant.

## Results

### Testosterone activates NFAT in cardiac myocytes

**Fig 1:**
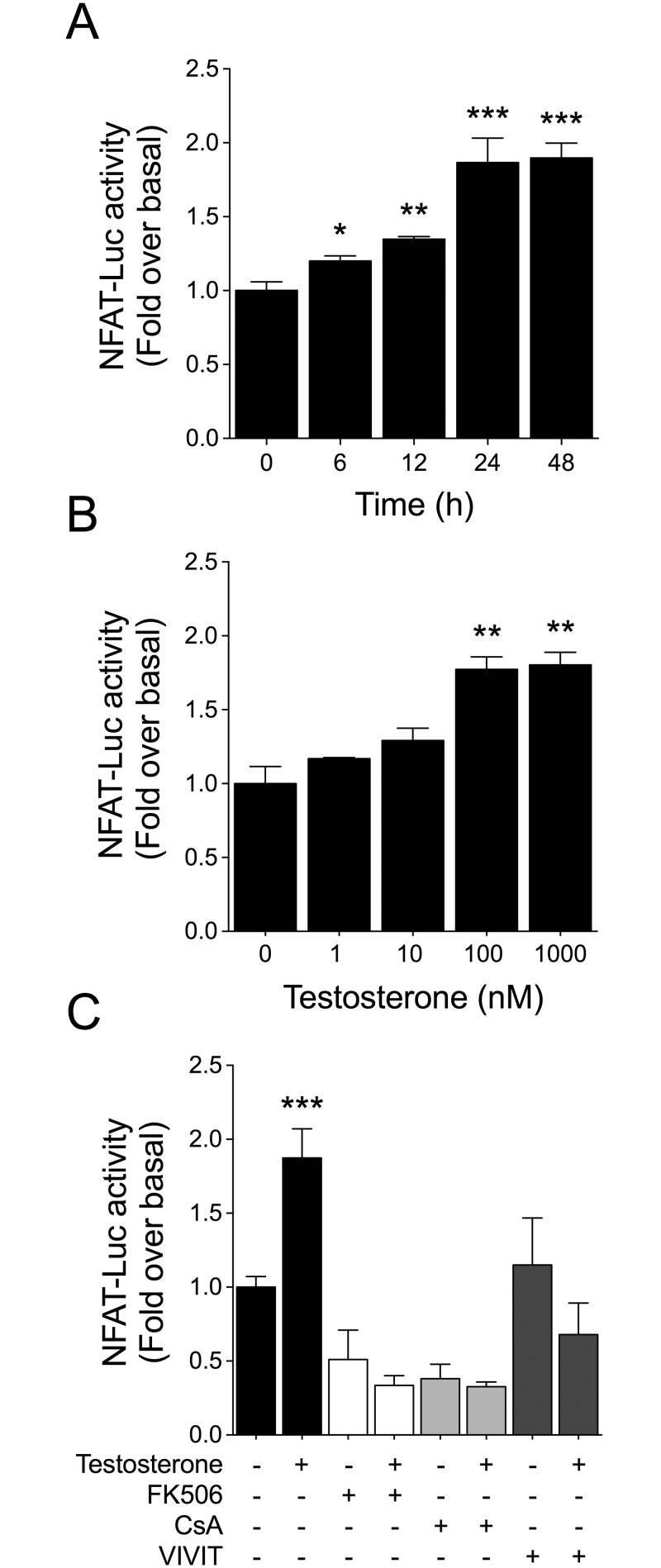
Testosterone activates NFAT in cardiac myocytes. NFAT activity was determined in cardiac myocytes transfected with a NFAT luciferase-reporter plasmid (NFAT-Luc) and normalized relative to *Renilla* luciferase activity in each sample. NFAT-Luc activity is expressed as fold induction relative to non-stimulated cells. (A) Cardiac myocytes were stimulated with 100 nM testosterone for 6, 12, 24, and 48 h. (B) Concentration-dependent effect of testosterone on NFAT-Luc activity. Cells were stimulated with 1, 10, 100, or 1000 nM testosterone for 24 h. Treatment with 100 and 1000 nM testosterone significantly increased NFAT-Luc activity as compared with non-stimulated cells. (C) Pretreatment of cardiac myocytes for 30 min with FK506 (1 μM), CsA (1 μM), or 11R-VIVIT (1 μM) prior testosterone stimulation (100 nM by 24 h) reduced NFAT-Luc activation. Values are presented as the mean ± SEM (n = 5 for each condition). *p < 0.05, **p < 0.01, and ***p < 0.001 *vs*. control non-stimulated condition.

NFAT is a well-described transcription factor implicated in cardiac hypertrophy. Previously, we showed that 100 nM testosterone induced hypertrophy in cardiac myocytes [7]. Thus, we initially used this concentration to investigate the time course of testosterone-induced NFAT activation. To examine the effect of testosterone on NFAT-mediated transcription, cardiac myocytes were transfected with a NFAT luciferase reporter plasmid (NFAT-Luc). Stimulation of cardiac myocytes with 100 nM testosterone increased NFAT-Luc activity between 6 and 48 h (Fig 1A). Next, to evaluate whether NFAT-Luc activity was dependent on testosterone concentration, cardiomyocytes were stimulated with 1, 10, 100, or 1000 nM testosterone by 24 h. NFAT-Luc activation increased in a concentration-dependent manner and peaked at 100 nM testosterone (Fig 1B).

NFAT activity is regulated through calcineurin [19], and testosterone induces intracellular Ca^2+^ increase in cardiac myocytes [6]. Thus, to test whether testosterone-dependent NFAT activation involves calcineurin, we incubated cardiac myocytes with FK506 (1 μM) or CsA (1 μM) prior to hormone stimulation, and found that both calcineurin inhibitors abolished NFAT-Luc activation induced by testosterone (Fig 1C). Additionally, specific NFAT-Luc activation was confirmed using 11R-VIVIT (1 μM), a cell-permeable peptide inhibitor that prevents NFAT dephosphorylation at its N-terminal region. As expected, treatment of cardiac myocytes with 11R-VIVIT blocked the increase in NFAT-Luc activity stimulated by testosterone (Fig 1C).

**Fig 2:**
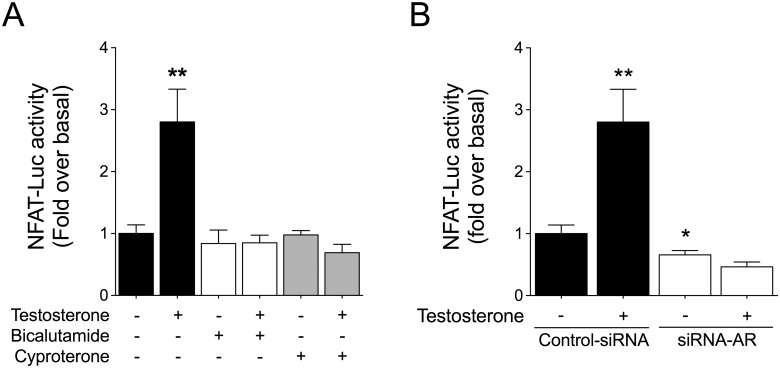
Testosterone-induced NFAT activation depends on androgen receptor. Cardiac myocytes expressing NFAT-Luc were pretreated with AR inhibitors. (A) Cells were pretreated for 30 min with bicalutamide (1 mM) or cyproterone (1 μM) before stimulation with testosterone (100 nM) for 24 h. (B) Cardiac myocytes were transfected with siRNA-AR (20 nM) or non-targeting siRNA as a control. AR downregulation abolished the increase in NFAT-Luc activity induced by testosterone. Values are presented as the mean ± SEM (n = 4 for each condition). *p < 0.05 and **p < 0.01 *vs*. control.

Testosterone exerts its biological effects largely by activating intracellular AR. To examine the participation of this receptor in NFAT-Luc activation, we used the pharmacological AR inhibitors bicalutamide (1 μM) and cyproterone (1 μM). Pretreatment of cardiac myocytes with the AR inhibitors abolished NFAT-Luc activity increase triggered by testosterone (Fig 2A). Similar results were obtained in cells transfected with siRNA targeting AR (siRNA-AR) (Fig 2B). Here, Western blotting analysis revealed that AR protein expression was decreased by ~51% in cells transfected with siRNA-AR (S1 Fig).

### Testosterone inhibits GSK-3β in cardiac myocytes

**Fig 3:**
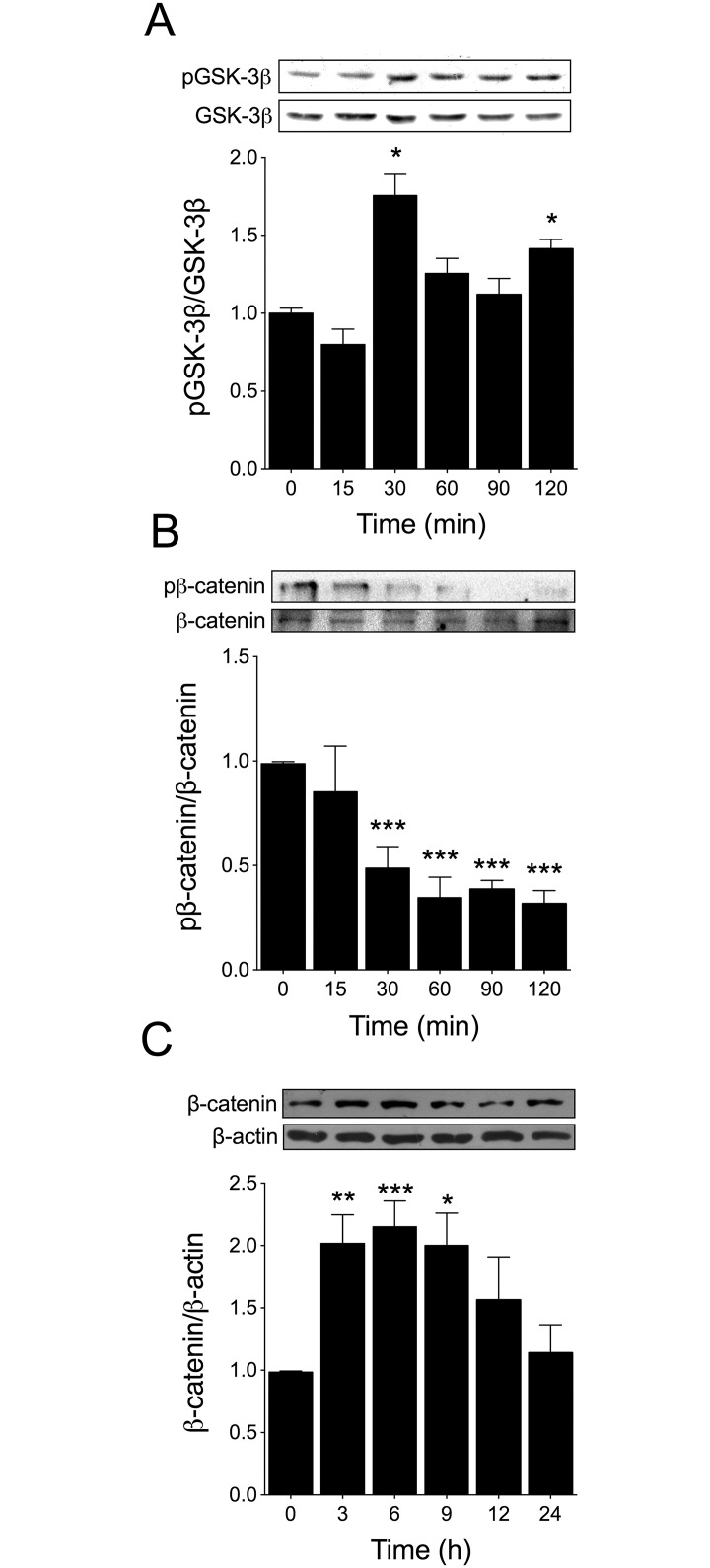
Testosterone inhibits GSK-3β in cardiac myocytes. Cardiac myocytes were stimulated with 100 nM testosterone for 15, 30, 60, 90, and 120 min and subjected to Western blot analysis to determine (A) GSK-3β phosphorylation (p-GSK-3β, Ser9) and protein levels (n = 5) and (B) β-catenin phosphorylation and total β-catenin protein levels (n = 4). The densitometric analyses show the ratio of phosphorylated versus total protein. Testosterone increased GSK-3β phosphorylation at Ser9 and decreased β-catenin phosphorylation at Ser33, Ser37, and Thr41 after 30 min of stimulation. (C) Cardiac myocytes were stimulated with 100 nM testosterone for 3, 6, 9, 12, and 24 h and total β-catenin protein accumulation was determined through Western blotting (n = 5). Values are presented as the mean ± SEM. *p < 0.05, **p < 0.01, and ***p < 0.001 *vs*. control non-stimulated condition.

It is well established that NFAT activity can be regulated by GSK-3β [24]. To investigate the role of GSK-3β in testosterone-induced NFAT activation, we first evaluated the phosphorylation levels of GSK-3β at residue Ser9 [38]. As shown in Fig 3A, stimulation with 100 nM testosterone increased GSK-3β phosphorylation after 30 min and 120 min. Next, we determined the levels of β-catenin phosphorylation and total β-catenin protein accumulation as markers of GSK-3β inhibition [26]. Testosterone suppressed β-catenin phosphorylation at Ser33, Ser37, and Thr41 starting 30 min after hormone stimulation (Fig 3B). Moreover, testosterone increased total β-catenin protein accumulation at 3–9 h after stimulation (Fig 3C).

### PI3K/Akt pathway is involved in testosterone-induced GSK-3β inhibition in cardiac myocytes

**Fig 4:**
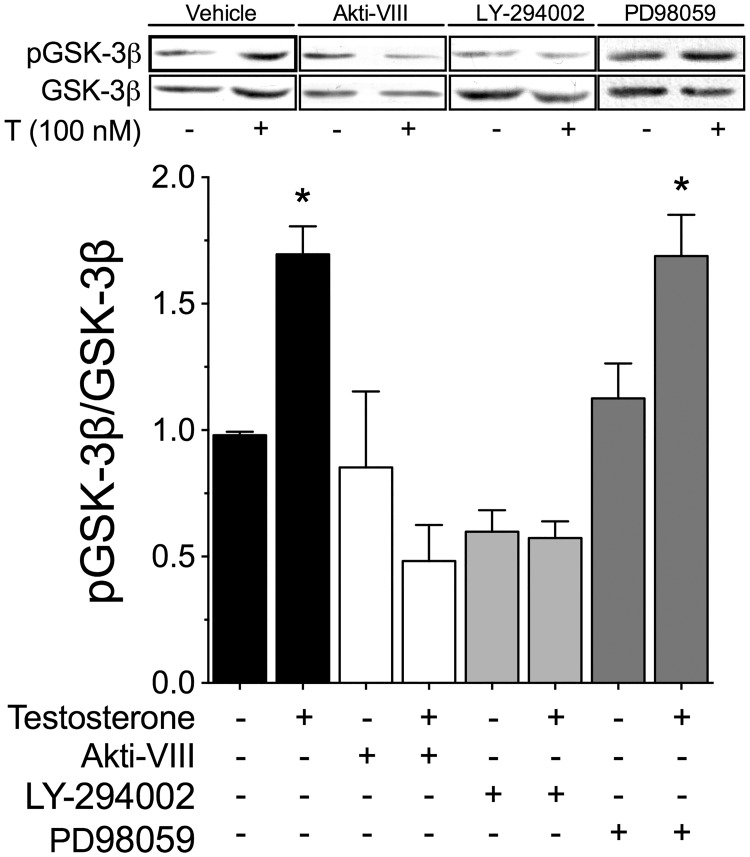
PI3K/Akt signaling is involved in GSK-3β inhibition. Cardiac myocytes were pretreated for 30 min with Akt-inhibitor VIII (Akti-VIII, 10 μM), LY-294002 (10 μM), or PD98059 (50 μM) prior to testosterone stimulation (100 nM) by 30 min. The densitometry results show the ratio of phosphorylated protein to total protein (n = 4 for each condition). Values are presented as the mean ± SEM. *p < 0.05 *vs*. control.

Among the upstream cascades known to influence GSK-3β, PI3K/Akt and MEK/ERK1/2 are the main pathways controlling the phosphorylation levels of GSK-3β at Ser9 [26–29]. To examine whether these pathways are involved in the phosphorylation of GSK-3β induced by testosterone, cardiac myocytes were pretreated for 30 min with Akt-inhibitor VIII (10 μM; an inhibitor of Akt), LY-294002 (10 μM; an inhibitor of PI3K), or PD98059 (50 μM; an inhibitor of MEK) prior to testosterone stimulation (100 nM, by 30 min). Whereas inhibition of the MEK/ERK1/2 pathway exerted no effect, PI3K/Akt inhibition blocked the GSK-3β phosphorylation induced by testosterone (Fig 4). These results suggest that activation of the PI3K/Akt pathway is involved in testosterone-induced GSK-3β phosphorylation.

### GSK-3β regulates NFAT activation induced by testosterone

**Fig 5:**
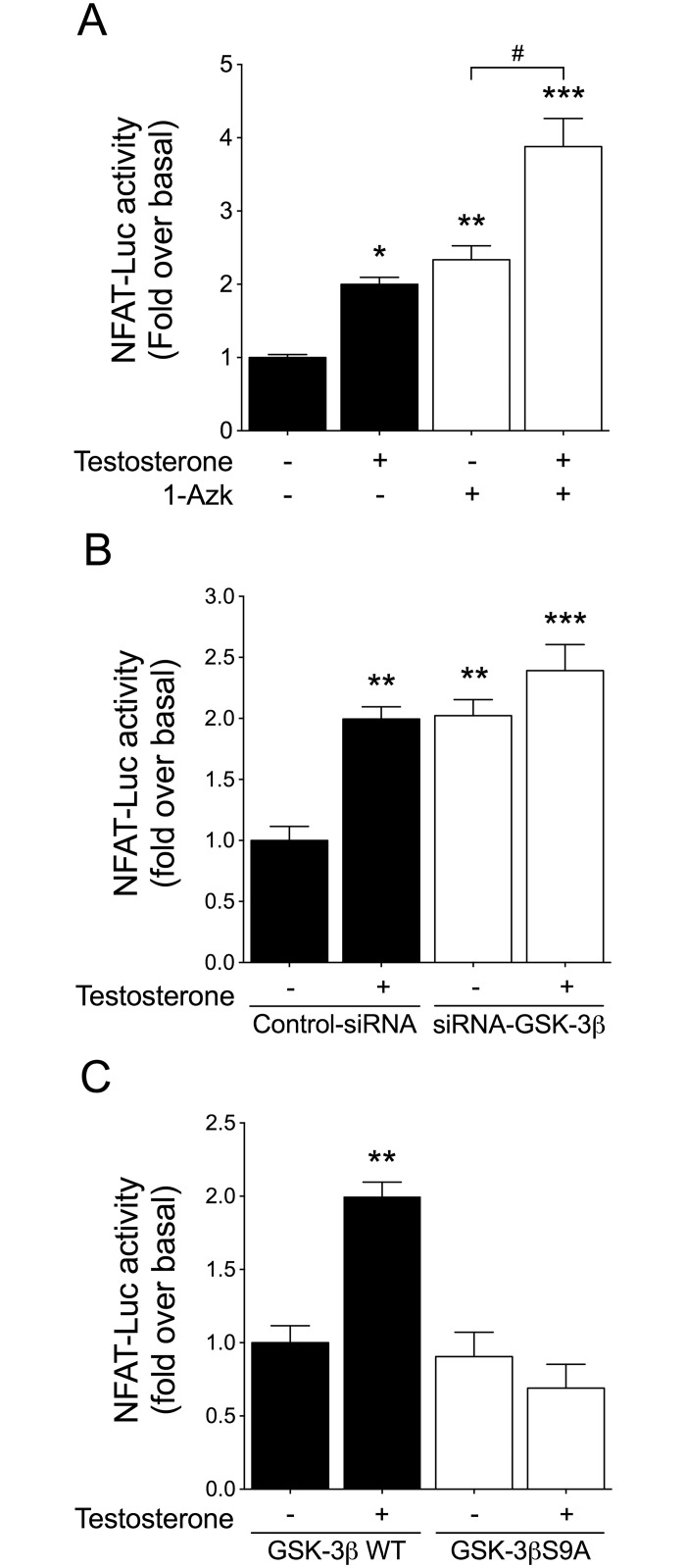
GSK-3β inhibition activates NFAT. Cardiac myocytes were co-transfected with NFAT-Luc and *Renilla* luciferase plasmids. (A) Cells were pretreated with 1-Azk for 30 min (1 μM) prior to 100 nM testosterone stimulation for 24 h. (B) Cardiac myocytes were transfected with either siRNA-GSK-3β or a non-targeted siRNA control and then stimulated with 100 nM testosterone for 24 h. (C) Cardiac myocytes were transfected with either GSK-3βWT or GSK-3βS9A, and then stimulated with 100 nM testosterone for 24 h (n = 6 for each condition). Values are presented as the mean ± SEM. *p < 0.05, **p < 0.01, and ***p < 0.001 *vs*. control; # p < 0.05 *vs*. 1-Azk.

To assess whether GSK-3β inhibition is involved in NFAT activation stimulated by testosterone, cardiac myocytes were pretreated with 1-azakenpaullone (1-Azk, 1 μM) or transfected with siRNA targeting GSK-3β (siRNA-GSK-3β). In cardiac myocytes transfected with siRNA-GSK-3β, the protein expression levels decreased by 59% as compared with cells transfected with the negative siRNA control (S2 Fig). GSK-3β inhibition with 1-Azk increased the basal activity of NFAT-Luc, and the subsequent stimulation with testosterone resulted in an additive increase in NFAT-Luc activity (Fig 5A). A similar effect was determined in cardiac myocytes transfected with siRNA-GSK-3β as compared with cells transfected with control siRNA (Fig 5B). To determine whether testosterone-induced NFAT activation involves GSK-3β inhibition, cardiac myocytes were transfected with a plasmid expressing a constitutively active form of GSK-3β (GSK-3βS9A) or a plasmid expressing wild-type GSK-3β (GSK-3βWT), which was used as control. Overexpression of GSK-3βS9A blocked the NFAT-Luc activity induced by testosterone (Fig 5C). These results suggest that testosterone increases NFAT activity through GSK-3β inhibition in cardiac myocytes.

### NFAT/GSK-3β signaling is involved in testosterone-induced cardiac myocyte hypertrophy

**Fig 6:**
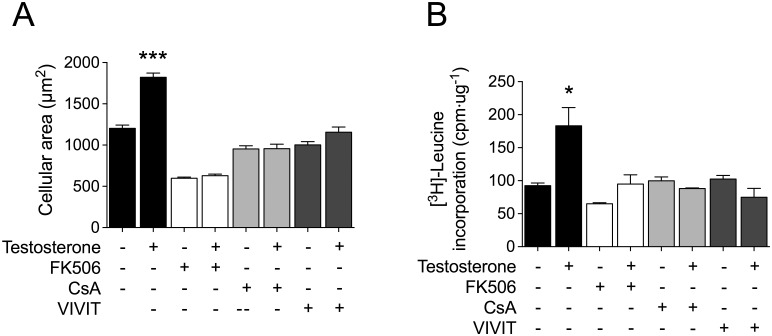
NFAT is involved in cardiac myocyte hypertrophy induced by testosterone. Cardiac myocytes were pretreated with FK506 (1 μM) or CsA (1 μM) or 11R-VIVIT (1 μM) and stimulated with 100 nM testosterone for 48 h. (A) For experiments involving cellular area measurement, the cardiac myocytes were incubated with CellTracker Green and visualized by confocal microscopy (n > 80 cells from 4 independent cell cultures). (B) Protein synthesis was determined based on [^3^H]-leucine incorporation. The data correspond to the ratio (basal counts/min)·μg^-1^ protein for each experimental condition (n = 6 for each condition). Values are presented as the mean ± SEM. *p < 0.05, and ***p < 0.001 *vs*. control non-stimulated condition.

**Fig 7:**
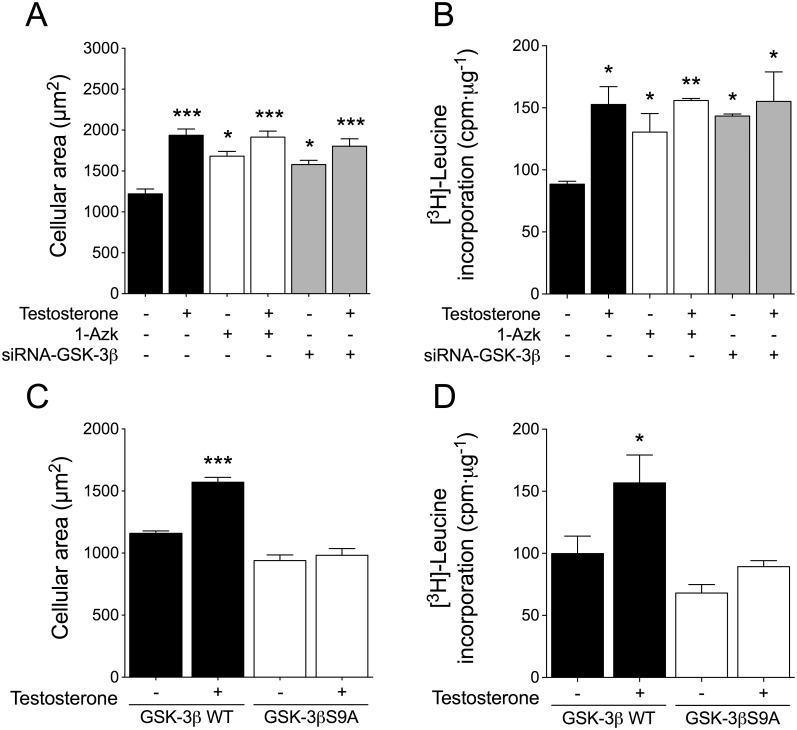
GSK-3β inhibition is involved in testosterone-induced cardiac myocyte hypertrophy. Cardiac myocytes were pretreated with 1-Azk (1 μM) or transfected with siRNA-GSK-3β (A and B, respectively), or the cells were transfected with GSK-3βWT or GSK-3βS9A expression plasmid (C and D, respectively) and then stimulated with 100 nM testosterone for 48 h. For cell size measurement, cardiac myocytes were incubated with CellTracker Green and examined by confocal microscopy (n > 80 cells from 4 independent cell cultures). Protein synthesis was determined by [^3^H]-leucine incorporation. The data correspond to the ratio (basal counts/min)·μg^-1^ protein for each experimental condition (n = 6 for each condition). Values are presented as the mean ± SEM. *p < 0.05, **p < 0.01, and ***p < 0.001 *vs*. control non-stimulated condition.

To ascertain whether GSK-3β/NFAT signaling cooperates with the AR pathway to induce cardiac myocyte hypertrophy, we first evaluated testosterone-stimulated changes in cell size and [^3^H]-leucine incorporation (as a measurement of cellular protein synthesis). Whereas treatment of cardiac myocytes with 100 nM testosterone for 48 h increased both cell size and [^3^H]-leucine incorporation (Figs 6 and 7), these hypertrophic effects of testosterone were prevented by FK506, CsA, and 11R-VIVIT (Fig 6A and 6B). Next, to investigate the functional effects of GSK-3β inhibition on cardiac myocyte hypertrophy, cells were pretreated with 1-Azk or transfected with siRNA-GSK-3β prior to testosterone stimulation (100 nM) for 48 h; the GSK-3β inhibition caused by the two treatments was found to increase the basal levels of both cell size and [^3^H]-leucine incorporation (Fig 7A and 7B). Testosterone stimulation in the presence of 1-Azk or siRNA-GSK-3β slightly increased both cell size and [^3^H]-leucine incorporation (Fig 7A and 7B), as compared with what was observed with the inhibitors applied alone. Furthermore, overexpression of GSK-3βS9A, but not GSK-3βWT, abolished the increase of both cell size and [^3^H]-leucine incorporation induced by testosterone (Fig 7C and 7D). These results suggest a role for the GSK-3β/NFAT pathways in androgen signaling in cardiac myocytes.

## Discussion

Currently, cardiac hypertrophy is widely accepted to involve both the activation of hypertrophic signaling and the inhibition of anti-hypertrophic pathways [39]. In this study, we determined that testosterone activates NFAT through calcineurin activation and GSK-3β inactivation, thereby inducing cardiac myocyte hypertrophy. Our findings are in accord with the underlying mechanism for testosterone action, which involves AR activation and regulatory signaling pathways [6, 8].

The biological actions of testosterone are mostly mediated by AR, which, under activation by testosterone, translocates to the nucleus and binds to specific androgen response elements (AREs) in the promoter regions of target genes [40]. Functional effects are elicited once AR is activated either directly or indirectly by interaction with specific coactivator proteins that regulate transcriptional responses [40]. In addition, current evidence indicates that testosterone activity is regulated through both AR-dependent and -independent mechanisms [9, 41]. NFAT/calcineurin signaling has been well established as a regulator of hypertrophic growth, and we here provide new insights regarding the mechanisms implicated in cardiac myocyte hypertrophy induced by testosterone. In this study, we determined that testosterone activates NFAT in a time- and concentration-dependent manner in cardiac myocytes. The transcriptional activity of NFAT is regulated tightly by intracellular Ca^2+^ through calcineurin [19], and we have previously shown that testosterone increases intracellular Ca^2+^ levels in cardiac myocytes, neuroblastoma cells, and skeletal muscle cells [6, 10, 42]. In cardiac myocytes, testosterone-dependent NFAT activation involves calcineurin, and we found that both FK506 and Cs abolished NFAT-Luc activation. Additionally, specific NFAT-Luc activation was confirmed using 11R-VIVIT, a cell-permeable peptide inhibitor that prevents NFAT dephosphorylation at the N-terminal region. Calcineurin dephosphorylates conserved serine residues in the N-terminal region of NFAT, which results in NFAT nuclear import [19]; NFAT then binds to its target promoters and activates the transcription of specific genes, either directly or in combination with other nuclear partners to orchestrate their biological effects [19]. Transgenic animals that express a constitutively active mutant of calcineurin or NFATc3 undergo cardiac hypertrophy [43]. A central role for Ca^2+^ signaling in cardiac hypertrophy was also revealed by studies on transgenic animals, in which overexpression of genes encoding components of Ca^2+^ signaling pathways caused hypertrophy [14, 44]. Furthermore, calcineurin inhibition by CsA and FK506 prevents androgen-induced growth in prostate cancer cells [21]. In a recent study, Qin et al. (2015) showed that testosterone and nandrolone administration in mice activated calcineurin—NFAT signaling and thus prevented muscle atrophy induced by denervation, which suggests that activation of Ca^2+^-dependent pathways are required for gene expression associated with androgen-induced muscle growth [20].

Dynamic regulation of NFAT activity allows the generation of specific cellular responses [17]. Here, we determined that inhibition of AR abolished NFAT activation induced by testosterone in cardiac myocytes. Transcriptional activation by androgens involves binding of the hormone-AR complex to specific ARE sites in the DNA [45]. The ARE-bound complex can induce gene transcription directly or can associate with co-regulators that cooperate with the transcriptional machinery [20]. The results of the present study support a role for NFAT in the transcriptional activity of AR signaling in cardiac myocytes. In LNCaP cells, AR induces AP-1 activity through generation of specific changes in the composition of the AP-1 DNA-binding complex [46], which are produced before the occurrence of androgen-mediated changes in cell growth [46], and NFAT contains AP-1 regions that mediate gene transcription by binding to DNA [16].

The calcineurin-dependent activation of NFAT can be regulated by nuclear kinases [14, 17] Phosphorylation mediated by GSK-3β promotes NFAT nuclear export and, subsequently, inhibition of its transcriptional activity [24]. The recognized isoforms of GSK-3, α and β, display Ser/Thr kinase activity and share 97% sequence homology in their catalytic domains. GSK-3β is a constitutively active Ser/Thr kinase whose initially identified role was the phosphorylation of glycogen synthase during glycogen production [26, 38, 47]. However, GSK-3β has now been implicated in several other key cellular processes, including cell-fate determination, metabolism, transcriptional control, and production of anti-hypertrophic effects [23, 48]. The diverse actions of GSK-3β depend on the type of stimuli and on GSK-3β phosphorylation status, intracellular localization, and interaction with other proteins that modulate its activity [38]. Upstream cascades as PI3K/Akt [26, 49] and ERK1/2 [28–30] modulate GSK-3β activity. To elucidate the mechanism involved in mediating the effects of testosterone on GSK-3β, we pharmacologically inhibited these signaling pathways. Whereas ERK1/2 inhibition produced no effect, inhibition of the PI3K/Akt pathway blocked the increase in the phosphorylation of GSK-3β at Ser9 induced by testosterone.

In cardiac myocytes, testosterone negatively regulates GSK-3β activity by 1) increasing the phosphorylation levels of GSK-3β at Ser9; 2) reducing β-catenin phosphorylation; and 3) reducing total β-catenin protein accumulation [32]. GSK-3β inactivation by androgens has also been observed in forebrain extracts from female rats, in which testosterone supplementation increased GSK-3β phosphorylation at Ser9 and decreased the phosphorylation at Tyr216 [50]. β-catenin and AR physically interact in prostate cells in response to testosterone. Moreover, a crosstalk between AR and the canonical Wnt signaling in neuronal cells has been described [51, 52].

A mechanism proposed for GSK-3β-dependent prevention of the hypertrophic growth of cardiac myocytes involves the phosphorylation and inhibition of NFAT [24]. Moreover, in cardiac myocytes, GSK-3β overexpression attenuated endothelin-1-induced hypertrophy by preventing nuclear translocation of NFAT [22]. GSK-3β controls NFAT nuclear residence by phosphorylating serine residues that mask the NLS region and promoting NFAT retention in the cytoplasm through interaction with 14-3-3 protein [53, 54]. Thus, GSK-3β inhibition promotes the nuclear residence of NFAT and thereby induces cardiac myocyte hypertrophy [19, 24, 26]. Our results indicate that NFAT is involved in the cardiac myocyte hypertrophy induced by testosterone. Moreover, the requirement of calcineurin and GSK-3β for NFAT activation by testosterone suggests a mechanism involving activation of hypertrophic pathways and inactivation anti-hypertrophic pathways in the mediation of androgen effects on cardiac myocytes. GSK-3β is detected in two states in the cytoplasm: in a free form or in a complex with APC/Axin to regulate the activity of the canonical Wnt/β-catenin pathway, which is associated with cell cycle control, survival, and cardiac hypertrophy [47, 55]. Here, we did not address the involvement of testosterone in APC/Axin/GSK-3β complex inhibition; however, activated AR has been shown to be capable of migrating to the nucleus to recruit β-catenin and thereby controlling the expression of genes associated with muscle differentiation [51], which suggests that testosterone may associate with the GSK-3β/β-catenin pathway [51, 52, 56]. This finding is intriguing because several reports have indicated that β-catenin/TCF/LEF pathway participates in cardiac myocyte growth [57]. Furthermore, β-catenin can be stabilized by pro-hypertrophic stimuli, and this is sufficient and necessary for inducing cardiac myocyte hypertrophy [58]. Our results showed that GSK-3β protein downregulation (siRNA-GSK-3β) or pharmacological inhibition (1-Azk) was sufficient for inducing cardiac myocyte hypertrophy, and that overexpression of a constitutively active mutant of GSK-3β (GSK-3βS9A) abolished the hypertrophy induced by testosterone. Furthermore, stimulation of cardiac myocytes with testosterone in the presence of GSK-3β inhibitors clearly induced an additional increase in protein synthesis, although a further increase in cell size was comparatively less evident. Other investigators have also reported observing such responses, and this indicates that cardiac myocytes exhibit a maximal and limited growth potentially due to the compensation between intracellular anabolic and catabolic processes under pro-hypertrophic stimulation [59].

In conclusion, in this study, we determined that testosterone modulates calcineurin/NFAT/GSK-3β and AR signaling pathways and thereby induces cardiac myocyte hypertrophy. These actions of testosterone might be associated with both the concentrations of the hormone and the differential activation of signaling pathways. Further investigation is required to elucidate the transcriptional mechanisms linked to androgen effects on cardiac cells.

## Supporting Information

S1 Fig Decrease of AR protein expression by siRNA-AR in cardiac myocytes. Cells were transfected with either negative siRNA control or siRNA-AR (20 nM) by 24 h. Western blot analysis shows that siRNA-AR reduced the expression of AR protein by ~51% with respect to control siRNA (n = 3). Values are the mean ± SEM. ** p<0.01 *vs*. control.
(TIFF)

S2 Fig Decrease of GSK-3β protein expression by siRNA-GSK-3β in cardiac myocytes. Cardiac myocytes transiently transfected with siRNA—GSK-3β exhibited a ~59% reduction of protein content with respect control siRNA-transfected cells. GSK-3β accumulation was determined by Western blot (n = 3). Values are the mean ± SEM. * p<0.05 *vs*. control.
(TIFF)
