# Supplementary figures and images for: GSK-3β/NFAT Signaling Is Involved in Testosterone-Induced Cardiac Myocyte Hypertrophy
Source: PLoS One. 2016 Dec 15;11(12):e0168255. doi: 10.1371/journal.pone.0168255 (PMC5158037; doi:10.1371/journal.pone.0168255)

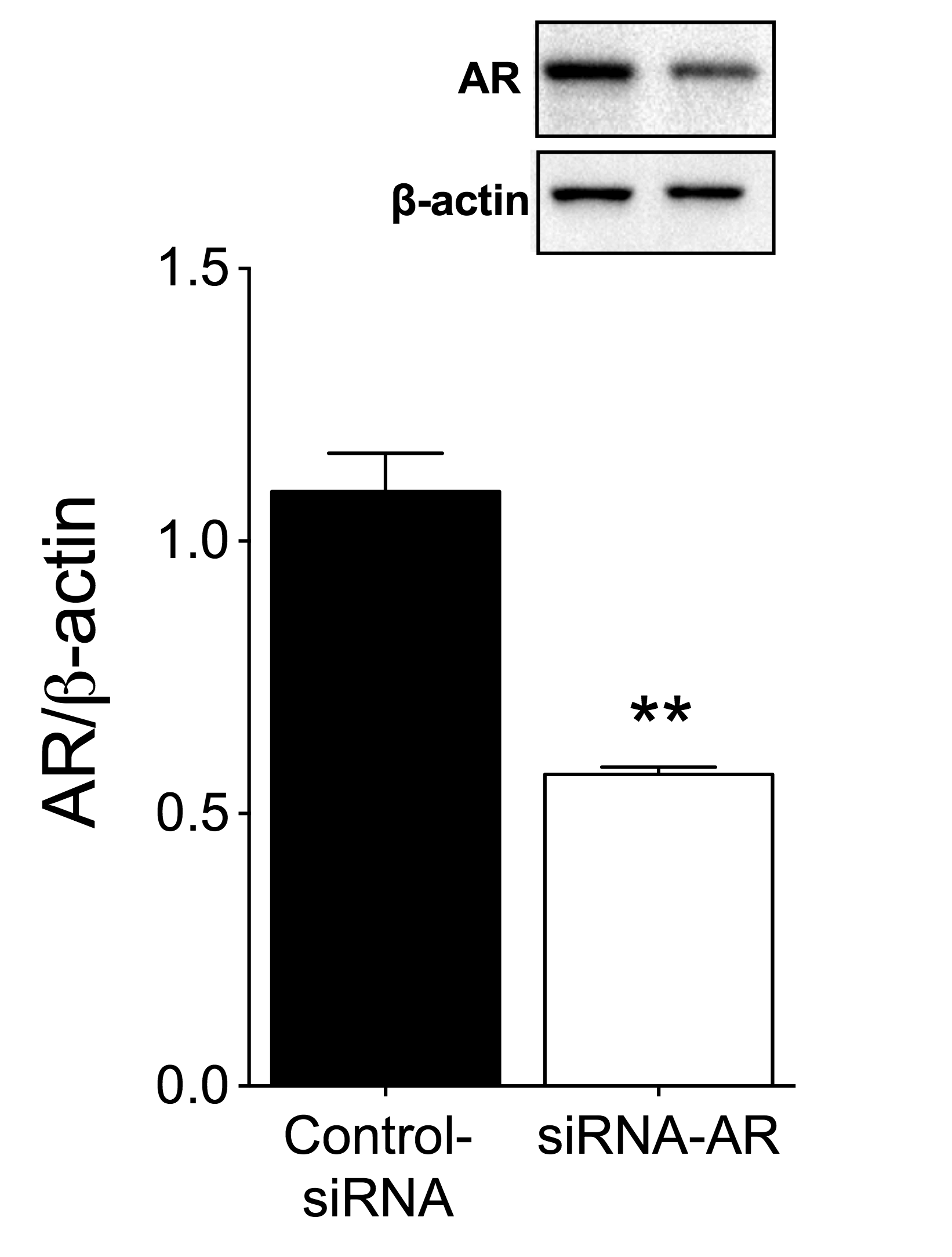

Supplement: S1 Fig — Cells were transfected with either negative siRNA control or siRNA-AR (20 nM) by 24 h. Western blot analysis shows that siRNA-AR reduced the expression of AR protein by ~51% with respect to control siRNA (n = 3). Values are the mean ± SEM. ** p<0.01 vs. control. (TIFF) [file pone.0168255.s001.tiff]

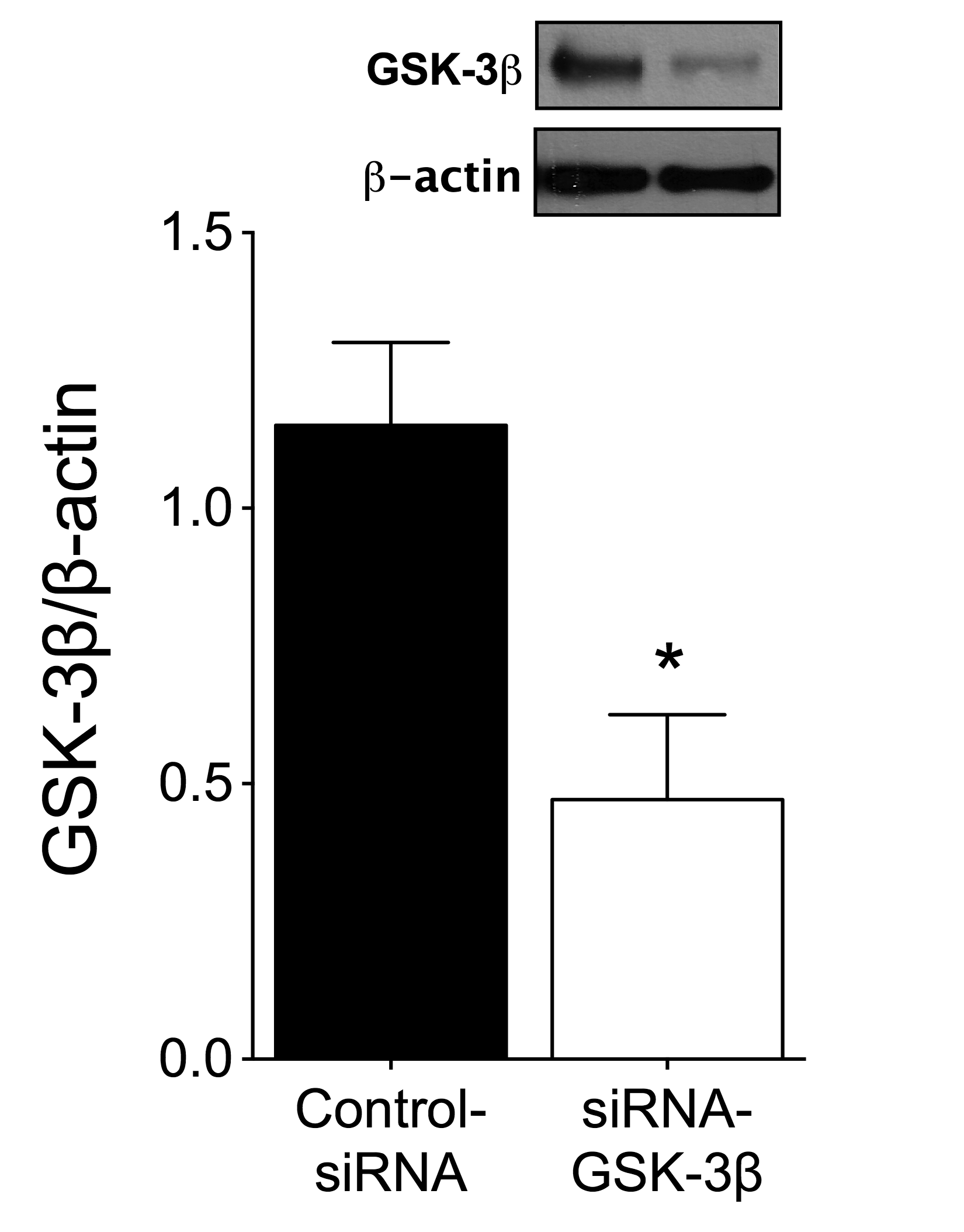

Supplement: S2 Fig — Cardiac myocytes transiently transfected with siRNA—GSK-3β exhibited a ~59% reduction of protein content with respect control siRNA-transfected cells. GSK-3β accumulation was determined by Western blot (n = 3). Values are the mean ± SEM. * p<0.05 vs. control. (TIFF) [file pone.0168255.s002.tiff]
